# Extracellular derived-exosomal micrornas in pancreatic cancer: investigating their diagnostic importance and potential targets for the prevention and treatment in pancreatic cancer

**DOI:** 10.3389/fonc.2025.1669213

**Published:** 2025-11-19

**Authors:** Rashid Mir, Ulfat Jan, Jameel Barnawi, Naseh A. Algehainy, Mohammed M. Jalal, Malik A. Altayar, Reema M. Almotairi, Tarig Ms Alnour, Syed Khalid Mustafa, Abdulaziz S. Al-Otaibi, Adel D. Althaqafy, Elham M. Alhathli, Salma Alrdahe, Mohammad Muzaffar Mir, Nada Zaki Sageer, Abdullatif Taha Babakr, Afaq Ahmad Khan

**Affiliations:** 1 Prince Fahad Bin Sultan Chair for Biomedical Research, University of Tabuk, Tabuk, Saudi Arabia; 2 Department of Medical Laboratory Technology, Faculty of Applied Medical Sciences, University of Tabuk, Tabuk, Saudi Arabia; 3 Genome diversity unit, University of Tabuk, Tabuk, Saudi Arabia; 4 Department of Chemistry, Faculty of Science, University of Tabuk, Tabuk, Saudi Arabia; 5 Faculty of Nursing, University of Taif, Taif, Saudi Arabia; 6 Department of Biology, Faculty of Science, University of Tabuk, Tabuk, Saudi Arabia; 7 Department of Clinical Biochemistry, College of Medicine, University of Bisha, Bisha, Saudi Arabia; 8 King Faisal Special Hospital, MOH, Makkah, Saudi Arabia; 9 Department of medical biochemistry, Faculty of medicine, Umm al-Qura University, Makkah, Saudi Arabia; 10 Department of Clinical Haematology, Sher-I -Kashmir Institute of Medical Sciences, Soura, Jammu & Kashmir, India

**Keywords:** exosomal microRNAs, pancreatic cancer, biomarkers, diagnosis, prognosis

## Abstract

Pancreatic cancer stands out as a deadly disease because patients receive late diagnosis and struggle with ineffective treatments. Exosomal microRNAs (miRNAs) that exist inside lipid bilayers help tumors grow and spread while making cells resistant to treatment and enabling cell-to-cell communication. Their ability to stay stable in body fluids makes them good candidates for early disease detection and treatment prediction tests. Research shows that miR-21, miR-17-5p, and miR-155 exosomal miRNAs help pancreatic cancer progress but also provide new targets for medical treatment. This review consolidates current evidence on the diagnostic, prognostic, and therapeutic potential of exosomal miRNAs in pancreatic cancer, integrating mechanistic insights into key signaling pathways such as PTEN/PI3Kγ, KRAS/MAPK, and TGF-β. Compared with previous reports, this work provides a comparative framework linking disease-specific exomiR profiles to other cancers, highlighting miR-21, miR-17-5p, miR-155, and miR-301a as central modulators. We further discuss methodological challenges, translational opportunities, and future directions in developing exosome-based diagnostics and miRNA-loaded therapeutic platforms. Understanding exosomal miRNA networks can pave the way for precision detection and targeted therapy in pancreatic cancer

## Introduction

Pancreatic cancer (PC) ranks as the seventh most prevalent source of cancer-related mortality worldwide, primarily due to infrequent diagnosis and scant treatment possibilities, leading to an alarming survival rate of just 9% over five years (1). The malignant tissue sample is most often acquired using invasive techniques like: endoscopic ultrasound-guided biopsy, endoscopic retrograde cholangiopancreatography (ERCP), brush cytology, or computed tomography (CT)-guided percutaneous biopsy (2, 3). Nevertheless, these techniques lack adequate sensitivity and specificity for early-stage disease detection. As a result, there is a great demand to identify new biomarkers that will enable early detection, effective treatment monitoring and accurate prognosis prediction in this unfortunate disease (4). Notable candidates for PC biomarker discovery are microRNAs (miRNAs), a class of small non-coding RNAs of approximately 19-23 nucleotides (5). The aberrant miRNA expression patterns have been reported in various malignancies including PC; in particular, such expression patterns can act as PC diagnostic and prognostic markers (6).

**Figure 1:**
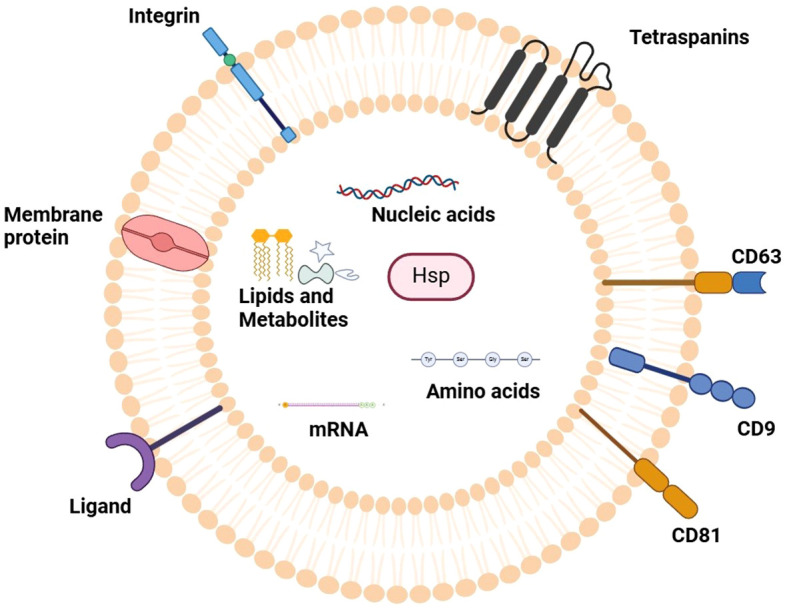
Representation of typical exosome structure. The figure outlines the defining characteristics of an exosome, which is a type of small extracellular vesicle with a membrane lipid bilayer. Relevant surface markers described above that characterize the vesicles known as exosomes are tetraspanins (CD9, CD63, CD81); they are positioned in the membrane. The internal cargo contains an assortment of bioactive compounds including proteins, messenger RNA (mRNA), microRNAs (miRNAs), and lipids which serve to modulate cellular communication, intercellular signaling and maturation of the target cells. This structural outline increases understanding of exosomes regarding their complex constituents and structures and their importance in biology under normal and disease conditions.

Exosomes, which are tiny extracellular vesicles with a size ranging between 30 and150 nm, are secreted by different cells including cancer cells into biological fluids like blood, urine, saliva, and even pancreatic fluid. They are formed from the endosomal pathways and transport bioactive compounds like protein, lipids, DNA, mRNA, and miRNAs between different cells (7). Exosomes maintain their stability and functionality through their lipid bilayer membrane which contains tetraspanins (CD9, CD63, CD81), heat shock proteins, and integrins. Exosomes take specific molecules from cells and deliver them to other cells to change how genes work and how cells signal while also affecting their immune response. PC tumors use exosomes to advance their growth while spreading to new areas and helping them avoid immune system detection. Exosomes show great potential as non-invasive tests because they remain stable in body fluids and contain tumor-specific information about pancreatic cancer (8, 9). The current review will focus on giving a critical and extensive summary of the available evidence on exosome-derived microRNAs (exomiRs) in pancreatic cancer (PC) and its diagnostic, prognostic, and therapeutic applications. Compared to previous reviews, which summarized mostly descriptive evidence, this manuscript incorporates recent mechanistic understanding of exosomal miRNAs regulation of oncogenic pathways (e.g., KRAS/MAPK, PTEN/PI3Kγ, and TGF-β) and therapy resistance. This work summarizes the available evidence across biofluids, cell types, and clinical stages to define the emerging trends, unresolved methodological concerns, and suggest translational approaches to clinical implementation. What is new about this review is not only that it combines exosomal miRNAs and pancreatic oncogenic signaling, but also that it takes a translational viewpoint, comparing recent molecular discoveries with possible diagnostic and treatment strategies specific to pancreatic cancer. The **Figure 1** illustrates the typical structure of an exosome, highlighting its lipid bilayer membrane, surface markers, and internal cargo, including proteins, mRNA, and miRNAs.

Detecting exosomal miRNAs (ExomiRs) provides a new opportunity for extracting possible biomarkers from biofluids (10). In the case of pancreatic cancer, there is often an overabundance of certain miRNAs, and their altered expression is associated with the onset and development of the disease (11). Furthermore, exosomes play a vital role in cell-to-cell communication by sending and delivering active molecules such as miRNAs (12). Exosomes derived from tumor cells can remodel the microenvironment and regulate neighboring cells behavior to promote tumor progression and form a supportive niche (13, 14). Therefore, exosomal miRNAs promise to be powerful PC biomarkers reflecting PC origin, diagnosis and PC progression by a simple biofluid assay. Pancreatic cancer (PC) is one of the deadliest cancers and ranks seventh in cancer related deaths worldwide (1). Pancreatic ductal adenocarcinoma (PDAC, the most common form of pancreatic cancer) has an overall 5-year survival rate of 12%. PDAC is generally associated with a poor prognosis, mainly due to late diagnosis; 80% of patients present with locally advanced or metastatic disease and the disease is also very aggressive and resistant to therapy (15). Although early detection and treatment of many cancers have made remarkable progress, no progress has been made in the case of PDAC, demonstrating the urgency of identifying novel diagnostic, prognostic and therapeutic targets for this disease (16).

Pancreatic cancer is associated with many risk factors, each of which falls under either changeable or unchangeable. In the case of risk factors that can be changed, smoking is the most important one since it almost triples the chances of getting pancreatic cancer. This increase in chances is associated with cancer-inducing substances contained in cigarette smoke that have the ability to cause changes to one’s DNA (17). A high-fat diet and obesity raise pancreatic cancer risk because fat tissue promotes inflammation and insulin resistance which promotes tumor growth. A sedentary lifestyle or being physically inactive makes metabolic problems worse. Chronic pancreatitis, characterized by long-term inflammation of the pancreas, significantly increases the malignant transformation (18, 19). Diabetes mellitus, particularly newly diagnosed or long-standing type 2 diabetes, is associated with pancreatic cancer, as insulin resistance and hyperglycemia can create a tumor-promoting environment (20, 21). Heavy alcohol consumption contributes to chronic pancreatitis and oxidative stress, increasing pancreatic cancer risk (22, 23). Prolonged exposure to industrial chemicals and pesticides has also been implicated, as carcinogens in these substances can induce DNA damage and oncogenic mutations (24).

**Figure 2:**
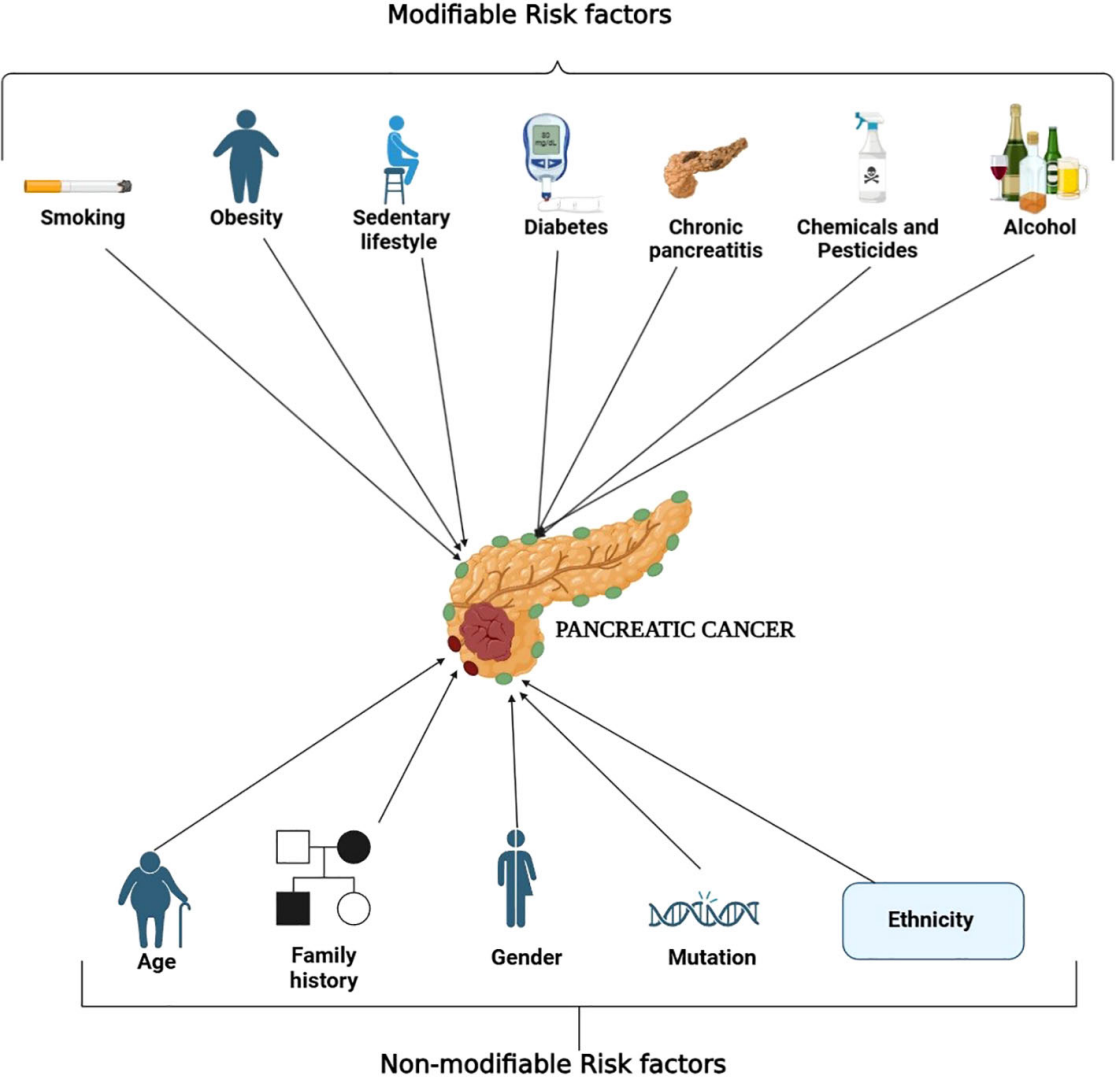
Overview of risk factors associated with the development of pancreatic cancer. The diagram separates the modifiable and non-modifiable risk factors associated with pancreatic cancer. Concerning modifiable risk factors, these include smoking, a diet high in fat, physical inactivity, obesity, chronic pancreatitis, diabetes mellitus, heavy alcohol consumption, and prolonged exposure to chemicals and pesticides. These risk factors are commonly associated with inflammation, insulin resistance, oxidative stress, and even stranding of oxidative DNA damage. On the other hand, age, family history, sex, inherited genetic mutations and ethnicity are classified as non-modifiable risk factors. The comprehensible classification of these categories assists in accuracy of detection and prevention measures.

Non-modifiable risk factors include increasing age, as most of the pancreatic cancer develops after age 65 because body cells suffer from too many years of genetic damage. When a direct relative experiences pancreatic cancer, it greatly increases your personal risk of developing the disease (17). Some genetic reasons along with demographic characteristics also raise the chances of getting pancreatic cancer (25). Inherited disorders like hereditary pancreatitis, mutations in BRCA1 and BRCA2, along with Lynch syndrome and Peutz-Jeghers syndrome are linked to inefficient DNA repair pathways and higher susceptibility to cancer due to increased genomic instability (26). Also, men are at greater risk than women, likely attributable to differences in lifestyle and sex hormones (27). Furthermore, African Americans seem to be at a disproportionately greater risk, and this may arise due to an interaction of genetic factors and the environment. Having complete knowledge of these risk factors helps us develop better ways to spot pancreatic cancer early while reducing risks for patients (28). **Figure 2** categorizes the risk factors for pancreatic cancer into modifiable and non-modifiable factors for better visualization.

A class of short, non-coding RNAs that post transcriptionally regulate target mRNAs, microRNAs (miRNAs) have been shown to play an important role in cancer development (29). Several cancers have been associated with changes in miRNA expression profiles that correlate with the initiation, progression and metastasis of these cancers (30). As oncomiRs or tumor suppressors dysregulated miRNAs can affect the proliferation, apoptosis, migration, and metastasis of cancer cells (31). Importantly, miRNAs can also be isolated from body fluids, such as plasma, serum, urine and saliva, and the expression of some miRNAs has been found to be associated with cancer progression, treatment response and patient survival, suggesting their potential as cancer biomarkers (32).

MicroRNAs are small RNA (∼22nt) non-coding RNAs that regulate gene expression post transcriptionally by binding to the 3′untranslated region of target mRNAs and repressing translation and/or inducing mRNA degradation (33). Tumor associated miRNAs can be oncogenic (oncomiRs), which promotes cancer hallmarks or tumor suppressive (oncomiRs), which blocks cancer hallmarks (34). The hallmarks of cancer can be induced by altered miRNA expression patterns, and in this context, miRNAs are promising as diagnostic markers (35). All types of cells secrete 30–150 nm extracellular vesicles, exosomes that transport proteins, lipids, mRNAs and miRNAs. MiRNAs can be released from donor cells into acceptor cells, and modulate their behavior, affecting progression and metastasis (36, 37). Tenascin-C in tumor derived exosomes in metastatic pancreatic cancer attenuates collagen gel mediated tumor cell apoptosis through the TGF-β pathway in non-cancerous stellate cells (38). Pancreatic cancer cell exosomes activate hepatic stellate cells and stimulate extracellular matrix deposition through Wnt paracrine signaling (39). PC cell derived exosomal miR-10b promotes migration and invasion in normal pancreatic ductal epithelial cells through HOXD10 targeting. PC cell exosomal miR-105 from PC cells enhances vascular permeability and metastasis of PC cells to the liver (40).

This review seeks to examine the role of exosomal miRNAs in the context of pancreatic cancer with an emphasis on their use as prognostic and diagnostic biomarkers, their contribution in tumor advancement and resistance to therapy, and the therapeutic perspective of targeting exosomal miRNAs. It attempts to demonstrate, based on available evidence, the importance and prospective uses of exosomal miRNAs in the efficacious diagnosis and tailored therapy of pancreatic cancer. This review is organized around a central question: How do exosomal miRNAs play a role in the diagnosis, prognosis, and therapeutic resistance of pancreatic cancer? This theme is represented in each of the sections that discuss molecular mechanisms, diagnostic biomarkers, and therapeutic applications to provide conceptual continuity. In order to put the biological and clinical significance of exosomal miRNAs into perspective, it is necessary to comprehend the state of the art in the pancreatic cancer diagnostics and treatment.

## Pancreatic cancer: overview and current challenges

Pancreatic cancer (PC) is a highly lethal malignancy and the fourth leading cause of cancer related mortality in the United states (41). Complex treatment methodologies and clinical strategies have not improved the prognosis of pancreatic cancer (42). The biology specific to pancreatic tumors, which are inhibited from spreading due to a dense fibrotic stroma, and lack early screening and specific/delicate symptoms until the cancer has advanced, contribute to this (43). Even with debulking surgical efforts combined with multi agent chemotherapy regimens, PC is extremely chemo resistant due to the exploratory and fibrotic nature of tumor growth (44). All forms of FDA approved monotherapy are also maintainantly resistant against PC. Treatment outcomes are augmented by the discovery of molecular therapeutic targets and the design of small molecules against such targets (45, 46). Chemoresistance is due to aberrant expression and mutation of specific genes and signaling pathways targeted by currently available drugs. In addition, innate and adaptive PC tumor mechanisms suppress the tumor’s ability to be attacked by the immune system (47, 48).

## Epidemiology and statistics

Pancreatic cancer (PC) is one of the malignancies with the worst prognosis, based on its late diagnosis. Only 12% survive 5 years, making the 5-year survival rate for all stages just 12% (49). According to GLOBOCAN 2024, pancreatic cancer accounts for approximately 2.6% of all new cancer cases and 4.8% of total cancer deaths worldwide, underscoring its disproportionate lethality. Exosome is a subtype of extracellular vesicles (EVs) with a size from 30 to 150 nm and a phospholipid bilayer structure (50). Exosomes are thought to be intercellular communicators, transferring between cells proteins, lipids, and nucleic acids (51). Recent studies show that tumor cells can induce the biogenesis and secretion of exosomes to form a tumor friendly immune microenvironment (52–54). Exosomes originating from pathologically altered parent cells have a distinct cargo which reflects the pathological state of the parent cells. Exosomal microRNAs (miRNAs) are more resistant to environmental stresses, and thus are potential noninvasive diagnostic and prognostic biomarkers of cancers, including PC, than other cargoes. These alarming epidemiological trends reinforce the need to explore molecular biomarkers such as exosomal miRNAs for early detection and better disease stratification.

## Pathophysiology of pancreatic cancer

**Figure 3:**
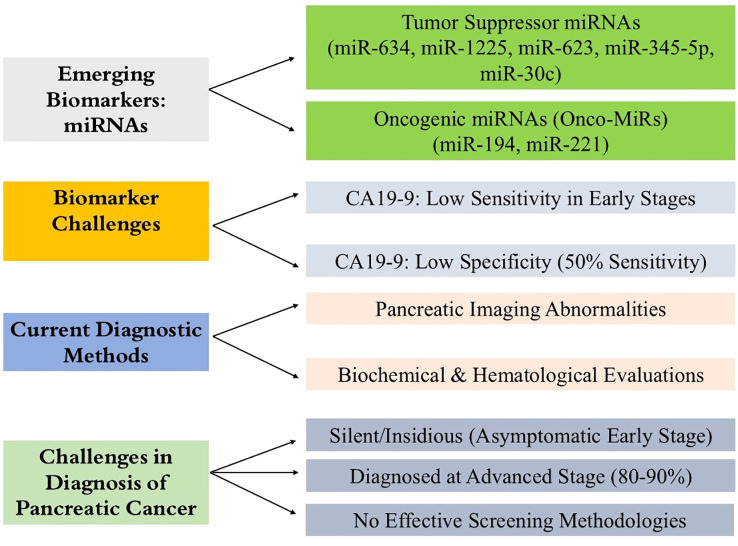
Diagnostic challenges in pancreatic cancer and the promise of miRNA biomarkers. The figure summarizes key barriers to early pancreatic cancer detection, including its asymptomatic progression and the limited sensitivity and specificity of current tools like CA19-9 and CEA. It also highlights the emerging potential of exosomal miRNAs as stable, noninvasive biomarkers with improved diagnostic accuracy for early-stage disease.

PC is usually diagnosed at an advanced stage, when treatment options are reduced (55). Presently, treatment does little to improve survival, underscoring the need for early detection of pancreatic cancer (56). And PC is silent and insidious, so patients may be asymptomatic in the early disease stage (57). Additionally, PC has no specific symptoms, and no effective screening methodology. Current methods of diagnosis of PC rely on the interpretation of pancreatic imaging abnormalities and the broad-spectrum evaluation of biochemical and hematological parameters (58). Currently, the diagnosis of PC is based primarily on the evaluation of imaging studies of the pancreas obtained through ultrasound, CT scans, magnetic resonance imaging (MRI) as well as a general assessment of metabolic and hematological factors with focus on the elevation of the tumor marker CA19-9 (**Figure 3**). Although these techniques are useful in discovering some form of abnormality in the pancreas, they lack the requisite specificity and sensitivity necessary to identify PC in its most amenable stages. This explains why a lot of people only seek medical attention when the disease has significantly advanced, which makes it harder to manage. There is a critical need for more accurate and easier diagnostic techniques for the condition, such as non-invasive biomarkers, capable of identifying the illness most reliably, which would enhance survival rates.

Multiple studies have shown a dysregulation of miRNAs in the development and progression of many different cancers including pancreatic cancer (59, 60). Several miRNAs have been identified with an oncogenic or tumor suppressive function (61). Newly reported tumor suppressor miRNAs in pancreatic cancer include miR-634, miR-1225, miR-623, miR-345-5p and miR-30c (62). On the other hand, onco-miRs (miRNAs with oncogenic functions) are miR-194 and miR-221. Pancreatic cancer altered expression patterns of these miRNAs make them promising diagnostic and prognostic indicators (59, 63).

## Effect of miRNAs on signaling associated with pancreatic cancer

Two of the most important KRAS signaling pathways that are affected by oncogenic activity include MAPK/MEK/ERK and PI3K/Akt. Both of these signaling pathways, inboard, are responsible for the control of growth, cell cycle, and cellular apoptosis, thus, are critical for the development and progression of PDAC (64). Also, many recent research findings highlight that a range of microRNAs (miRNAs) precisely control these oncogenic pathways through the modulation of tumor suppressor genes and oncogenes at the molecular level. These functions of miRNAs have the potential to inhibit or promote malignancy depending on the circumstantial context and the target genes’ semantics (65). Beyond the extensively studied miR-21 and miR-155, several other miRNAs including miR-181a, miR-196a, miR-221/222, and miR-210 have emerged as regulators of critical oncogenic cascades. miR-221/222 enhance invasion through the STAT3/MAPK axis, miR-181a targets SMAD4 to promote EMT, whereas miR-210 contributes to hypoxia adaptation and metabolic reprogramming in PDAC cells.

In particular, miR-29c is noted to be significantly downregulated in PDAC tissues. This phenomenon is attended by the upregulation of MAPK1, thus contributing to further access cellular proliferation and invasion via activating the downstream MAPK/ERK signaling cascade (66). Also, miR-98-5p - a tumor suppressive – was noted to be down regulated in PDAC contributing to the overexpression of MAP4K4. The increased MAP4K4 levels also support the activation of MAPK/ERK thus enhancing the PDAC cells’ proliferation, invasion, and migration *in vitro (*67).

Beyond tumor-suppressive miRNAs, certain oncogenic miRNAs, such as miR-21, have been proposed to contribute towards the progression of PDAC. miR-21 is known to stimulate EGFR signaling through downstream effectors by oncogenically targeting the negative regulator, Sprouty RTK signaling antagonist 2 (Spry2). This interaction further promotes downstream activation of MAPK/ERK and PI3K/Akt pathways, ultimately enhancing cell proliferation and tumor growth (68).

Several noteworthy examples of such miRNAs exist, which are capable of targeting multiple oncogenic pathways at once. For instance, miR-24-3p contributes towards the aggressiveness of PDAC by targeting LAMB3 and Anti-Silencing Function 1B (ASF1B). miR-24-3p is known to inactivate LAMB3 resulting in the activation of PI3K/Akt signaling axis, and simultaneously interacting with ASF1B drives epithelial mesenchymal transition (EMT) which is a pivotal process in metastasis (69).

Additionally, more recent investigations have noted tumor suppressive function of miR-30d. In a 2021 study, separate research groups noted low expression of miR-30d was associated with poor prognosis in PDAC patients. miR-30d acts on malignant phenotypes by silencing transcriptional oncogenes such as RUNX1 and SOX4, which leads to diminished expression of more aggressive traits (70). Interestingly, one prior study demonstrated that RUNX1 binds to the promoter region of miR-93 and inhibits its transcription, creating a feedback loop. This is important because miR-93 is known to suppress EMT, invasion, and migration in PDAC cells, indicating potentially intricate regulatory circuits involving miRNAs and their transcriptional regulators (71).

## Current diagnostic and prognostic methods

Pancreatic cancer patients often lose weight and develop new onset diabetes, but currently employed standard noninvasive tests such as CT image analysis and endoscopic ultrasound (EUS) are not effective for diagnosis as the tumor size (< 2 cm) is small at the early stage (72). However, among invasive procedures endoscopic retrograde cholangiopancreatography (ERCP) can be performed to obtain bile duct tissues to investigate for mutations but this approach is not simple because bile duct obstruction is typically absent in early-stage PC (73). Currently, screening and monitoring of PC relies on blood test of carbohydrate antigen 19-9 (CA19-9), the best validated PC biomarker, but its high false negative and positive rates (74). A class of small (19-25 nucleotides) non-coding RNAs, microRNAs (miRNAs) recognize complementary sequences in target mRNAs and either induce translational repression or mRNA degradation. They control about 30 percent of the human protein coding genes and are central to regulating many cellular processes (75). It is now emerging evidences that miRNAs play a role in the development and progression of cancers by controlling the expression of many oncogenes and tumor suppressors in an orchestrated fashion (76). Upregulated miRNAs in PC associated with inflammation include the most studied miRNA in PC, miR-21, and miR-155, as well as newly reported tumor suppressor miRNAs negatively regulated by oncogenic KRAS, miR-634 and miR-1225, respectively (77–79). In human tissues, pancreatic cancer is associated with downregulation of miR-95, -186, -217, -218, and -888 while upregulation of miR-10b, -21, -23b, -155, and -196 is observed (80–83). Therefore, aberrant miRNA expression patterns in pancreatic cancer tissues and biofluids might be exploited to distinguish pancreatic cancer patients from healthy individuals. Blood miRNAs are regarded as robust biomarkers for diagnosis, prognosis, and monitoring of pancreatic cancer (84).

## Existing treatment options and limitations

Because pancreatic cancer is so lethal and the seventh leading cause of cancer-related mortality in the world, in part, because of its poor prognosis. There is still early diagnosis as a major problem in clinical practice, and the existing biomarkers for early detection of pancreatic cancer are still imperfect (49). miRNAs are small, non-coding RNAs that post transcriptionally regulate gene expression by binding to complementary sequences in the targets mRNAs and leading to translational repression and/or mRNA degradation; therefore, they represent new classes of potential novel biomarkers for early pancreatic cancer detection (85). Since exosomal miRNAs have been detected in biofluids such as blood, urine, and saliva, many studies aimed to understand the roles of exosomal miRNAs in various types of carcinomas (86). By 2030, pancreatic cancer is projected to be the second leading cause of cancer related death. The disease is rapidly progressive, extends widely and early metastasizes, and is highly lethal (87, 88). Curative resection in pancreatic cancer patients is best treated with surgical treatment. Unfortunately, only a small fraction of patients (about 15-20%) are diagnosed with resectable pancreatic cancer; the majority come to us with locally advanced or metastatic disease (89). To better understand the diagnostic and therapeutic challenges in pancreatic cancer, the **Table 1** provides a concise summary of the key issues and emerging solutions:

**Table 1:** Key challenges and emerging solutions in pancreatic cancer.

Category	Challenges	Emerging Solutions
Epidemiology	High mortality rate (5-year survival: 9%); late-stage diagnosis in 80–90% of cases.	Early detection efforts through advanced biomarkers like miRNAs.
Diagnostic Limitations	Lack of specific early screening tools; CA19-9 sensitivity limited to advanced stages (79%).	Exploration of exosomal miRNAs as non-invasive diagnostic tools.
Therapeutic Limitations	Limited resectability (15–20% of cases); chemoresistance; low survival post-surgery (20%).	Development of targeted therapies and immunotherapies; focus on KRAS-targeting agents.
Biomarkers	CA19-9 lacks specificity (50% of patients with PC do not show elevated levels).	Validation of blood-based miRNAs as robust biomarkers for prognosis.
Current Treatments	Surgery followed by Gemcitabine or Capecitabine often shows modest success.	Investigations into small molecules and immune checkpoint inhibitors.

Following surgery, most patients receive adjuvant therapy, usually a combination of the chemotherapeutic agent Gemcitabine and the radiation sensitizer Capecitabine. However, these treatment options are often ineffective, and pancreatic cancer is generally resistant to most available therapeutic options. Although several novel therapeutic options, including targeted therapy or immunotherapy, have been developed, no new treatment has yet been approved for clinical use (90). Increasing evidence implicates dysregulated miRNAs in chemoresistance and therapeutic failure. Overexpression of miR-21, miR-155, and miR-23a suppresses PTEN and PDCD4, reducing gemcitabine sensitivity, whereas restoration of miR-34a or miR-143 re-sensitizes PDAC cells to treatment. Experimental delivery of tumor-suppressive miRNAs via exosomes or lipid nanoparticles is emerging as a promising adjunct to conventional regimens.

## Exosomes: biology, biogenesis, and functional composition

Exosomes are nano-sized extracellular vesicles (30-150 nm) which are released to the extracellular environment by most cell types. Mature endosomes are therefore multivesicular bodies formed from the inward budding of the plasma membrane and endocytosis (91). Exosomes were reported to package some proteins (MHC class I and II molecules) and RNAs (mRNAs and miRNAs) and then transfer them to adjacent or distant cells (92). Exosomes are critical for intercellular communication or crosstalk to deliver bioactive molecules, including protein, lipid, RNA, and DNA, to recipient cells to influence their physiological or pathological behavior (93). To provide a concise summary of exosomes’ biology and functions, the **Table 2** highlights key aspects of their structure, biogenesis, and roles:

**Table 2:** Key Features, Functions, and Applications of Exosomes.

Aspect	Details	References/Examples
Introduction	Nano-sized extracellular vesicles (30-150 nm) released by most cell types; formed via inward budding of plasma membrane and endocytosis.	Discovered during reticulocyte maturation; carry proteins (e.g., MHC), RNAs (mRNA, miRNA).
Biogenesis	1) Early endosomes formed by plasma membrane invagination. 2) Maturation into MVBs, creating ILVs with ESCRT machinery and ceramide lipids. 3) Secretion as exosomes.	Depends on cellular states (normal/pathological).
Composition	Contain lipids, proteins, mRNA, miRNA. ExomiRNAs influence processes like cancer progression and immune responses.	miRNA-301a (activates M2 macrophages via PTEN/PI3Kγ); miRNA-1246 (activates NF-κB pathway).
Role in Communication	Facilitate intercellular communication by transferring bioactive molecules (e.g., proteins, lipids, RNA, DNA) to distant or adjacent cells.	Enable paracrine signaling without direct cell-cell contact.
Therapeutic Potential	Non-immunogenic nature and protective cargo packaging make exosomes promising for gene therapy.	Challenges: Efficient miRNA loading into exosomes (solubility, charge factors).
Diagnostic Potential	ExomiRNAs serve as biomarkers with higher diagnostic accuracy than traditional markers.	5-exomiRNA signature (85% accuracy vs. 67% for CA19-9).

Understanding exosome biogenesis is essential to understand their use as biomarker or therapeutic agents. Generally, exosomes are produced and secreted in a three-step process. Below is a visual summary of the exosome biogenesis process, highlighting the three main steps involved in their formation and secretion.

1. Invagination of the plasma membrane occurs early endosomes with proteins and RNAs (94).
2. Some endosomal proteins are modified by ubiquitin, in which case the early endosomes are matured into late endosomes or multivesicular bodies (MVB). In addition to their content of intraluminal vesicles (ILVs), unique to MVBs, MVB formation is induced by activation of the endosomal-sorting complex required for transport (ESCRT) machinery in a protein-ubiquitin dependent manner and accumulation of ceramide lipids, sphingomyelin, and cholesterol (94, 95).
3. MVBs can either fuse with lysosomes and ILVs are degraded, or can fuse with the plasma membrane to secrete exosomes. The size and number of exosomes secreted from a cell are dependent on the state of the cell (e.g. normal or pathological) (94, 96).

Almost all cells can secrete exosomes containing lipids, proteins, mRNA and miRNA, which are dependent on the cell type and microenvironment (97). MiRNA can be released from cells into exosomes, or ‘exo-miRNAs’, and these exo-miRNAs have accumulated significant evidence for roles in cancer initiation and development (98). For example, exosomal miRNA-301a activated the PTEN/PI3Kγ pathway, and promoted M2 polarization of macrophages derived from hypoxic pancreatic cancer cells (99). Likewise, hypoxic pancreatic cancer cells were shown to secrete exosomal miRNA-1246 that activated the NF-κB pathway in non-tumor cells to induce the release of IL-6 and IL-8, to promote EMT of pancreatic cancer cells (98). Previous studies have demonstrated that exomiRNAs can be novel biomarkers drawn from blood serum example a 5 exomiRNA signature that can classify healthy individuals and pancreatic cancer patients with 85% accuracy (100). But traditional serum biomarker CA19-9 was found to classify in PC patients (101). It was found that pancreatic cancer cells secrete miRNA-155 exosomes to activate fibroblasts and promote desmoplasia (102). Additionally, exosomes are non-immunogenic, and protect the biological cargo and are therefore ideal gene therapy systems. However, major hurdles in exosomes reaching the clinic include efficient miRNA loading into exosomes, which are influenced by solubility and charge (103). The ability of exosomes to selectively package and deliver miRNAs underlies their diagnostic and therapeutic potential in pancreatic cancer, forming the foundation for subsequent sections of this review.

## MicroRNAs: biogenesis, processing, and gene regulation

MicroRNAs (miRNAs) are short, non-coding RNA molecules (~19–25 nucleotides) that regulate gene expression at the post-transcriptional level (104). They serve as key modulators of several cellular processes, including proliferation, differentiation, apoptosis, and metabolism. Aberrant miRNA expression has been reported in many human diseases, particularly cancer, where upregulation of oncogenic miRNAs (oncomiRs) such as miR-21 and miR-155 or downregulation of tumor-suppressive miRNAs such as miR-30d contributes to tumorigenesis (76). In pancreatic cancer, where late diagnosis and therapy resistance remain major challenges, miRNAs are being investigated as promising non-invasive biomarkers because of their stability and detectability in biofluids (105, 106). Combination with other molecules such as proteins further enhances reliability of biofluid microRNA profiling as a means of accurate diagnosis of pancreatic cancer (107, 108). Because pancreatic cancer is an aggressive disease with a poor prognosis, its deadly potential makes it an important candidate for biomarker discovery. There are several genomically encoded cultivated miRNAs in different families, characterized by sequence similarities. Alteration of miRNAs dysregulation leads to changes in their target genes and the pathological conditions such as cancer (109). Several pancreatic cancer (PC) behaviors have been shown to be attributable to emerging evidence of miRNA involvement. Being stable, feasible, and accessible, miRNAs can serve as biomarkers for diagnosis, prognosis, and monitoring of pancreatic cancer (106). There is no doubt about the critically ill need for early detection of pancreatic cancer. This, however, is hampered by the absence of specific symptoms and effective screening methods. The low sensitivity and specificity of current screening methods based on the combination of risk factor assessment and laboratory blood tests for the early detection of pancreatic cancer have been demonstrated (110). In recent years, genomic, transcriptomic, proteomic, and metabolomic researches have reported various potential pancreatic cancer biomarkers. Biomarker discovery technologies are from high throughput omics-based profiling to small targeted investigations. A number of pancreatic cancer biomarkers have been discovered, progressed to validation studies in independent cohorts, and some are on the path to commercialization (111).

The biogenesis of miRNAs follows a multistep process. miRNA genes are transcribed by RNA polymerase II as long primary transcripts (pri-miRNAs), which are subsequently cleaved in the nucleus by the Drosha–DGCR8 complex into precursor miRNAs (pre-miRNAs). These are exported to the cytoplasm by Exportin-5 and further processed by the RNase III enzyme Dicer into short double-stranded miRNA duplexes (112, 113). One strand, known as the guide strand, is incorporated into the RNA-induced silencing complex (RISC), while the complementary passenger strand is degraded (114). The mature miRNA–RISC complex then recognizes complementary sequences in the 3′ untranslated regions (3′-UTRs) of target messenger RNAs (mRNAs), leading to translational repression or degradation of the target transcript (11). Consequently, exosomal miRNAs may be novel noninvasive biomarkers and therapeutic targets for cancer.

Pancreatic cancer is generally a poor prognosis disease due to late diagnosis and resistance to treatment. Pancreatic cancer is usually diagnosed at early stages when the clinical symptoms are often not obvious and the cancer is not evident on imaging technique. Pancreatic cancer is therefore often misdiagnosed with chronic pancreatitis even with the help of imaging techniques (115). Early detection of pancreatic cancer is currently not possible using current serum biomarkers. Pancreatic cancer is usually diagnosed at advanced stages, at which time surgical resection is infeasible. The use of exosomal microRNAs (miRNAs) as promising diagnostic and prognostic biomarkers for pancreatic cancer has been intensively investigated (116). Exosomal miRNAs are uniquely stable in biofluids and can withstand harsh condition such as high enzymatic activity, extreme pH and high temperature. In human tissues and biofluids, extensive profiling studies have identified many differentially expressed exosomal miRNAs in pancreatic cancer versus normal or benign controls (117). In addition, exosomal miRNAs can be targeted to prevent the development and progression of pancreatic cancer as therapeutic targets. Preclinical studies have reported several candidates as exosomal miRNA targets for the treatment of pancreatic cancer (118, 119).

MicroRNAs (miRNAs) are small non-coding RNA molecules that are important in the post-transcriptional regulation of gene expression. miRNAs are 19–25 nucleotide long molecules that bind to complementary sequences within target mRNAs and inhibit their translation, deadenylate and degrade the target transcripts, and are important in regulating a plethora of important biological processes including cell development, cell cycle, proliferation, differentiation, apoptosis and stress responses (120). The pathogenesis of human cancers has been associated with deregulation of miRNA expression. The functional role of the miRNA let-7 was first reported as a tumor suppressor in lung cancer and the first report of a miRNA able to regulate cancer pathogenesis (76). Thereafter, several studies have demonstrated that several miRNAs are overexpressed or downregulated in pancreatic cancer and exosomes. Various studies shows that the functional miRNAs within exosomes of donor cells can be transferred to target cells, and can modulate the expression of target proteins, were the first to provide evidence that exosomes contain functional miRNAs (119, 121–124). From then on, numerous studies have examined the functional roles of exosomal miRNAs in many human diseases including cancer.

## Exosomal microRNAs in pancreatic cancer

This section synthesizes current evidence on exosome-derived miRNAs in pancreatic cancer, focusing on their disease-specific expression patterns and molecular roles. For most cancers, death rates have fallen since 1990, except for PC, which has the greatest increase. There were about 495,773 new pancreatic cancer cases and 466,003 related deaths in the world in 2020 (125). PC is usually diagnosed at advanced stages and often inoperable because of early subtle symptoms and poor screening methods. At present, surgical resection remains the only curative option, but only 15-20% of patients at diagnosis are eligible (126). Due to lack of current systemic chemotherapy options and their ineffectiveness, urgent developments of early detection, prevention, and treatment are needed (127).

miRNAs are biogenetically transcribed by pol II or pol III to yield primary miRNA transcripts that are coarsely processed by an enzyme complex containing the RNAse III enzyme Drosha and the double stranded RNA binding protein Pasha (128). miRNAs are dysregulated in pancreatic cancer, and are implicated in the development and progression of cancers (129). Different cancers have been found to contain miRNAs with oncogenic or tumor suppressing functions. Well studied oncomiRs in pancreatic cancer include miR-7, miR-21, and miR-155 and newly reported tumor suppressor miRNAs include miR-634, miR-1225, miR-623, miR-345-5p, miR-30c, miR-194 and miR-221 (62). (**Table 3**) miRNAs are attractive candidates as diagnostic and prognostic indicators because their expression in pancreatic cancer is altered (130). Due to the complex and context dependent role of miRNA in cancer, the role of miRNA in PC is often not fully studied.

**Table 3:** Key insights on pancreatic cancer and exosomal miRNAs.

Category	Details	Examples/Key Findings
Pancreatic Cancer Overview	PC cases and deaths in 2020: 495,773 and 466,003, respectively. Often diagnosed at advanced stages; resectable in only 15-20% of cases.	Current systemic therapies remain ineffective; urgent need for early detection and novel therapeutic approaches.
MicroRNAs (miRNAs)	Small (22-nucleotide) non-coding RNAs regulating 70% of human mRNA transcripts.	Dysregulated miRNAs include oncomiRs (e.g., miR-7, miR-21, miR-155) and tumor suppressors (e.g., miR-634, miR-345-5p).
Circulating miRNAs	Found in plasma; potential non-invasive biomarkers for pancreatic cancer.	Around 20 circulating miRNAs show elevated levels in PC patients.
Exosomal miRNAs	miRNAs carried in exosomes, crucial for intercellular communication and tumor microenvironment regulation.	miR-17-5p and miR-21 are elevated in PC. Hypoxic PC cells secrete miR-301a-enriched exosomes, polarizing M2 macrophages via PTEN/PI3K signaling.
Tumor Microenvironment	Exosomal miRNAs modulate immune responses, EMT, and therapy resistance.	PSC-derived miR-21-enriched exosomes activate RAS/ERK signaling. NK cell exosomes with miR-3607-3p downregulate IL-26, suppressing progression. PC cells use miR-194-5p to repair DNA damage post-radiotherapy.

miRNAs are critical players of cellular processes including tumor development in humans and control nearly 70% of mRNA transcripts. More than a dozen human cancers, including PDAC, have been linked to altered miRNA expression (131). There is a lot of miRNA expression studies that show that there is a significant difference in miRNA expression between normal pancreatic ductal cells and pancreatic cancer cells. The past few years have seen circulating miRNAs investigated as potential biomarkers, and 20 circulating miRNAs have been identified that are found to be elevated in patients with pancreatic cancer. Promising diagnostic tools for pancreatic cancer are plasma-based miRNA panels (59). A large body of literature has been devoted to studying exosomal miRNAs, a key cargo of exosomes, in pancreatic cancer and other malignancies.

Researchers in 2013 found higher levels of miR-17-5p and miR-21 exosomes in the blood of patients who had pancreatic cancer. Scientists found that the exosomal miRNAs named in that study actively participate in pancreatic cancer progression across multiple development phases (132). NK cell exosomes loaded with miR-3607-3p reduce disease progression through lower IL-26 levels in the body (133). Pancreatic stellate cells produce exosomes containing high levels of miR-21 that cancer cells consume to switch their cell structure from EMT through RAS/ERK pathway activation. Research shows that pancreatic cancer cells can use specific exosomal miRNAs such as miR-194-5p to pause their cell division and make repairs to damaged DNA before regrowing after radiotherapy treatment (134) (**Table 3**).

**Figure 4:**
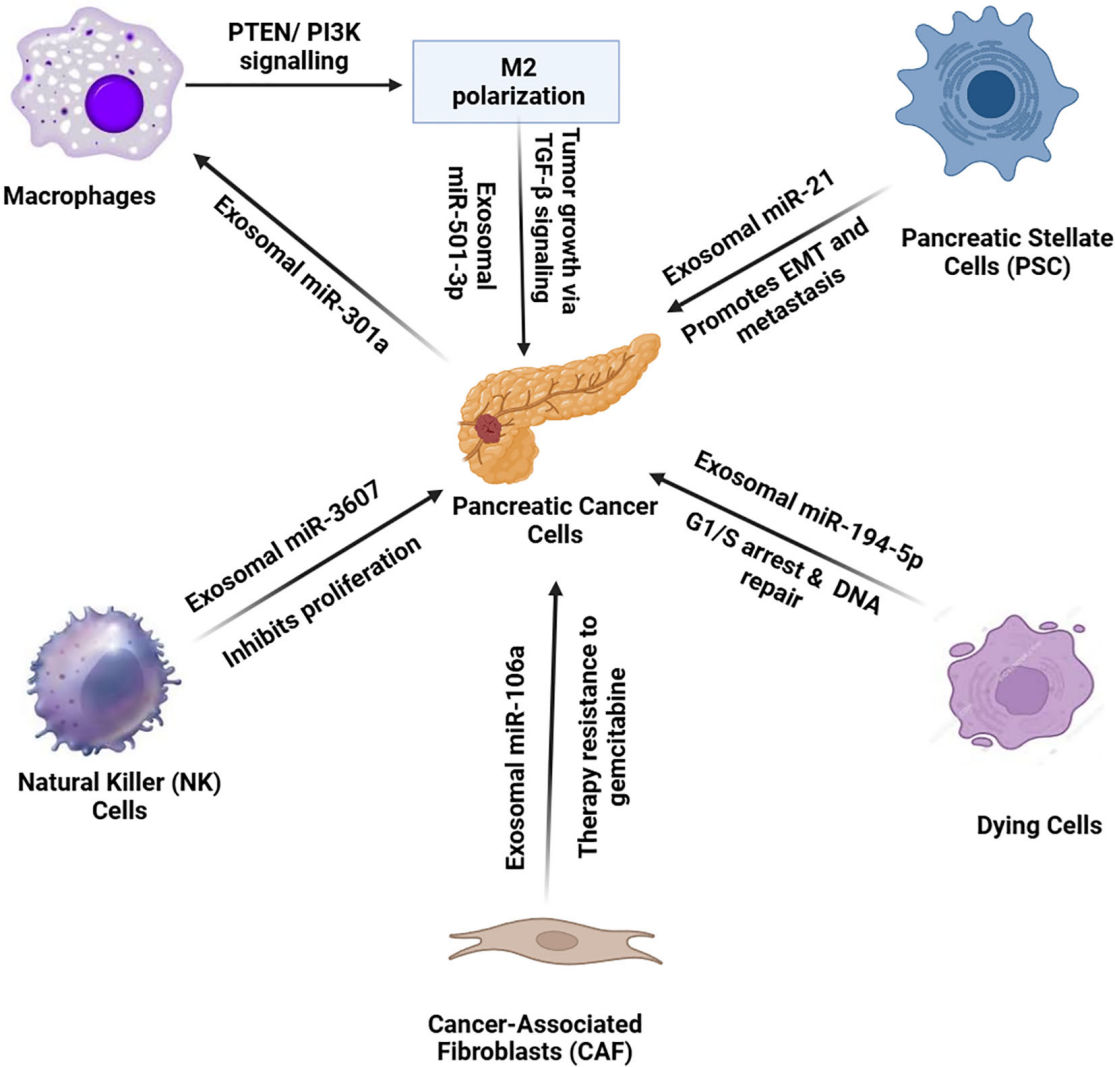
In the pancreatic cancer tumor microenvironment, various cell types communicate with pancreatic cancer cells through exosomal miRNAs, influencing tumor progression and therapy resistance. Cancer-associated fibroblasts (CAFs) release exosomes enriched with miR-106a, inducing resistance to gemcitabine therapy. Pancreatic stellate cells (PSCs) secrete miR-21-containing exosomes, promoting EMT and metastasis. Natural killer (NK) cells release miR-3607, inhibiting cancer cell proliferation. Dying cancer cells release exosomes with miR-194-5p, causing G1/S cell cycle arrest for DNA repair. Under hypoxic conditions, pancreatic cancer cells release miR-301a-enriched exosomes, inducing M2 macrophage polarization. Polarized macrophages then secrete miR-501-3p, activating TGF-β signaling to enhance tumor growth. This complex network of exosomal miRNAs facilitates tumor progression and resistance mechanisms.

When pancreatic cancer cells face oxygen deprivation, they produce exosomes filled with miR-301a that turn macrophages into M2 cells by triggering PTEN/PI3K signaling. The macrophages use exosomes loaded with miR-501-3p to stimulate TGF-β signaling which drives tumor advancement (99). **Figure 4** presents the exosomal transfer of tumor microenvironment miRNAs in their interactions.

## Exosomal miRNAs as diagnostic biomarkers for PDAC

Many different miRNA biomarkers have been studied for pancreatic cancer over time, but their variability makes them difficult to use in practice. The multiple forms of these circulating miRNAs as free molecules or bound to proteins or inside exosomes along with their origin from multiple cell types creates difficulty in detecting them (106). Exosomal miRNAs provide a better detection option because they stay intact and protected inside lipid bilayers. Since these biomarkers come from tumor cells, they show tumor changes and help doctors detect diseases (135). To summarize the diagnostic potential of exosomal miRNAs, the **Table 4** provides key examples and their uses:

**Table 4:** Exosomal miRNAs in pancreatic cancer: diagnostic, prognostic, and therapeutic biomarkers.

Source	miRNAs	Use
Serum	miR-17-5p, miR-21	Diagnostic; prognostic; recurrence
	miR-1246, miR-4644, miR-3976, miR-4306	Diagnostic
	miR-10b, miR-21, miR-30c, miR-181a; miR-let7a (down)	Diagnostic
Plasma	miR-1246, miR-196a	Diagnostic
	miR-483-3p, miR-451a	Prognostic; recurrence
Blood	miR-16a, miR-196a	Diagnostic
Pancreatic Juice	miR-21, miR-155	Diagnostic; prognostic
Saliva	miR-1246, miR-4644	Diagnostic
Portal Vein	miR-4525, miR-451a, miR-21	Prognostic; recurrence
PCC	miR-23b-3p, miR-339-5p, miR-222	Prognostic
	miR-155, miR-27a, miR-212-3p	Gemcitabine resistance; immune suppression
	miR-301a, miR-194-5p	PTEN/PI3K signaling; prognosis
M2 Macrophages	miR-501-3p	TGF-β signaling; prognosis
NK Cells	miR-3607-3p	Prognostic
PSC	miR-210	Gemcitabine resistance
BM-MSC	miR-126-3p, miR-1231	Suppress development
UC-MSC	miR-145-5p	Suppress progression
CAF	miR-146a, miR-106b	Gemcitabine resistance

Comparative Landscape of Exosomal miRNAs in Pancreatic and Other Cancers A comparative perspective underscores both shared and disease-specific exosomal miRNA signatures across malignancies. For instance, miR-21 and miR-155 are ubiquitously upregulated in pancreatic, breast, and lung cancers, reflecting their generalized oncogenic role. However, miR-1246 and miR-301a exhibit relatively higher specificity for pancreatic cancer, mediating macrophage polarization and therapy resistance. **Table 5** summarizes the most reported exosomal miRNAs across key cancer types, outlining their diagnostic and functional implications.

**Table 5:** Comparative landscape of exosomal microRNAs (miRNAs) across pancreatic and other cancers: functional roles and clinical relevance.

Cancer Type	Key Exosomal miRNAs	Functional Role	Diagnostic/Prognostic Relevance
Pancreatic cancer	miR-21, miR-155, miR-301a, miR-17-5p	Oncogenic; therapy resistance (PTEN/PI3Kγ, TGF-β)	Diagnostic & prognostic
Breast cancer	miR-10b, miR-21	Metastasis, invasion	Prognostic
Lung cancer	miR-23b-3p, miR-210	Hypoxia adaptation	Diagnostic
Colorectal cancer	miR-200c, miR-1246	EMT regulation	Prognostic

Studies have discovered that pancreatic cancer detection can be supported by analyzing certain miRNAs found in exosomes. When measuring exosomal miRNA levels, researchers found that using a combination of miR-17-5p, miR-21, miR-1246, and miR-196a can help diagnose pancreatic cancer (132). The detection of pancreatic cancer reached 100% accuracy when analyzing miRNAs that were both increased (miR-10b, miR-21, miR-30c, and miR-181a) and decreased (let7a) (136). Research shows that measuring miR-483-3p in plasma exosomes helps doctors tell pancreatic cancer apart from IPMN and similar diseases (137).

Scientists have not yet found one unique set of exosomal miRNA markers for pancreatic cancer because different labs process samples in different ways and use different testing methods. We need uniform testing methods to make these findings reliable and usable in medical practice. When we add exosomal miRNA results to CA 19-9 measurements, we can better detect pancreatic cancer at an earlier stage (138).

## Exosomal miRNAs as prognostic biomarkers for PDAC

Exosomal miRNAs are important for how pancreatic cancer develops and how well patients do. Exosomal miRNAs guide gene regulation in nearby cells to control how blood vessels develop, how the immune system responds, and cancer cell spreading (139). In particular, research shows that higher levels of miR-451a in plasma exosomes are linked to cancer coming back, and miR-21 and miR-221 help pancreatic cancer cells, PSCs, and CAFs talk to each other and support tumor growth (140). When released in exosomes, the specific miRNAs miR-23b-3p, miR-339-5p, and miR-222 encourage cancer cells to multiply, travel, and invade other tissues (141–143). Pancreatic cancer cells, when exposed to oxygen deficiency, send enriched miR-301a-filled exosomes that reprogram immune cells called macrophages and make the cancer more likely to spread (144).

On the other hand, certain exosomal miRNAs act to prevent tumor growth. BMSC exosomes contain miR-126-3p that acts against ADAM9 to stop cancer cells from advancing. When BMSCs and umbilical cord mesenchymal stromal cells release miR-1231 and miR-145-5p, they stop pancreatic cancer from spreading (145, 146).

Exosomal miRNAs also predict therapy responses. When patients take lapatinib and capecitabine, higher levels of miR-221 in their body predict how well the drugs will work, as well as their resistance to treatment (132). The exosomal miRNAs miR-146a and miR-106b from cancer-associated fibroblasts promote gemcitabine resistance by regulating transcription factors and signaling pathways. The research demonstrates that exosomal microRNA levels can help predict patient treatment outcomes and drug resistance patterns (147).

## Exosomal miRNAs in the treatment of PDAC

Exosomes show great potential as drug carriers because they are safe for the body and help load therapeutic molecules. Researchers create exosomes to transport siRNAs or miRNAs that target KRAS mutations found in 90% of pancreatic cancer patients (148). Research with mice proves that exosomes delivering siRNA that targets KRAS-G12D can stop tumor progression and increase mouse survival times (149).

Exosome-delivered microRNAs could help doctors fight cancer in patients whose treatments fail. Exosomal miR-7 helps stop pancreatic cancer from growing and invading tissues by blocking MAP3K9, but cancer growth is boosted by miR-182 through its control of β-TrCP2 (132, 150, 151). We can improve how well treatments work by putting these miRNAs inside exosomes. We need additional studies and testing in clinical settings to confirm how exosomal miRNAs can be used best to treat pancreatic cancer.

A clinical trial (NCT04636788), launched in late 2020, aims to identify exosomal miRNA biomarkers for the early detection of pancreatic cancer. This ongoing study is recruiting both patients and healthy controls, with a target enrollment of 102 participants. Each participant will provide 12 mL of venous blood, and small RNAs—including miRNAs—will be analyzed using next-generation sequencing. The primary outcome measures are sensitivity and specificity of candidate biomarkers, while secondary measures include patient survival outcomes.

In 2017, engineered exosomes loaded with siRNAs targeting KRAS-G12D—a common mutation in PDAC—was tested in a mouse model. These clinical-grade exosomes, derived from KRAS-G12D-mutant mesenchymal stem cells, successfully downregulated the expression of mutant KRAS-G12D, resulting in prolonged survival with no significant toxicity (149, 152). These promising preclinical results led to the initiation of a Phase I clinical trial (NCT03608631) to evaluate the safety and efficacy of this strategy in PDAC patients harboring the KRAS-G12D mutation (153).

Additionally, preclinical studies have shown that specific miRNAs may serve as therapeutic cargo for engineered exosomes. Overexpression of miR-7, which targets MAP3K9, significantly inhibited tumor growth in pancreatic cancer models (154). In contrast, miR-182—known to target the cell cycle regulator β-TrCP2—has been implicated in promoting tumor proliferation and migration (155). Other miRNAs such as miR-205 and miR-182 are also being explored for inclusion in exosomal delivery systems (155). These findings suggest that loading tumor-suppressive miRNAs like miR-7 into engineered exosomes could represent a promising strategy for the targeted treatment of pancreatic cancer.

## Potential therapeutic targets

The increased serum levels of exosomal miR-17-5p and miR-21 was the baseline of the first study demonstrating diagnostic value of exosomal miRNAs in PC (156). Over the years, more research has been conducted, with many other exosomal miRNAs with diagnostic usefulness being discovered. For instance, exosomal miR-1246, miR-4644, miR-3976, and miR-4306 have all been documented to be elevated in PC patients (157). A multi-marker signature containing miR-10b, miR-21, miR-30c, miR-181a, as well as downregulated miR-let 7a, markedly surpassed classic exosomal markers like glypican-1 (158). Moreover, exosomal miR-451a is showing promise as a predictor for advanced disease and recurrence. Previous studies, emphasizes the presence of significant amounts of mir-196a and mir-1246 in PC exosomes and their elevation in plasma from patients with localized illness (159). Additionally, exosomal miRNAs from other sources such as pancreatic juice (miR-21, miR-155), saliva (miR-1246, miR-4644), and portal blood (miR-4525, miR-451a, and miR-21) have demonstrated potential for diagnostics as well as prognostics (132).

Exosomal communication between cancer cells and fibrotic tissue cells results in the upregulation of oncogenic miR-21 and miR-221 in pancreatic cancer cells because of cross talk with cancer-associated fibroblasts (CAFs) or pancreatic stellate cells (PSCs) (160). At the functional level, exosomal miR-23b-3p is known to enhance proliferation, migration, and invasion in various diseases. Likewise, miR-339-5p and miR-222 are associated with enhanced invasive potential. Hypoxia-induced exosomal miR-301a is known to foster the M2 macrophage phenotype via PTEN/PI3Kγ signaling, thereby enhancing metastatic potential (161). Exosomal miR-501-3p, derived from M2 macrophages, has the opposite effect of activating TGF-β signaling in PC cells and thus, drives tumorigenesis. However, not all exosomal miRNAs have an oncogenic potential.

They are also involved in immune evasion. For example, tumor-derived miR-212-3p, transported to dendritic cells, silences the RFXAP gene which is responsible for the expression of MHC class II molecules and antigen presentation on the cell surface. Although some progress has been made, the mechanisms controlling miRNA packaging, delivery, and functional activity within recipient cells remain elusive. More studies focusing on the architecture of exosomal communication and their movement is needed in order to use them in therapy.

Exosomal miRNAs are also important in PC chemoresistance, particularly with regard to gemcitabine. Exosomes from CAFs Snail and miR-146a release during gemcitabine exposure can induce resistance in cancer cells after being taken up (162). Similarly, miR-155 is upregulated in exosomes from gemcitabine-treated cancer cells. This miRNA confers resistance by downregulating DCK, an enzyme that activates gemcitabine. Resistance is reversible by inhibiting miR-155 or by overexpressing DCK, demonstrating therapeutic potential. CAF-derived exosomal miR-106b also contributes to gemcitabine resistance through TP53INP1 downregulation. Moreover, gemcitabine-resistant pancreatic cancer stem cells (PCSCs) secrete exosomal miR-210, which mediates transfer of resistance to more sensitive cells (163, 164).

Despite the rising evidence for exosomal miRNA involvement in chemoresistance, many gaps still exist. Understanding the mechanisms behind selective miRNA encapsulation and uptake, as well as pertinent resistance-associated miRNAs, will be essential. Filling these gaps may enhance therapeutic approaches that aim to alleviate drug resistance in PC.

## Challenges in developing miRNA-based therapies

Creating miRNA-based therapies for pancreatic cancer faces major barriers that must be met to realize their clinical value. One key issue is the effective delivery of therapeutic miRNAs to the target tumor cells. MiRNAs, due to their unstable nature, face rapid degradation by nucleases in circulation, necessitating protective delivery systems such as lipid nanoparticles, viral vectors, or exosomes. Specialized lipid nanoparticles, viral vectors, or exosomes can provide stability and ensure uptake on a structural level; however, these systems are unfortunately unable to avoid off-target effects, delivering medicine to areas outside of the tumor.

A different challenge presents itself in the selective targeting of oncogenic or tumor-suppressing miRNAs. Although a wide range of miRNAs are considered to be dysregulated in cancer, almost all of them seem to have pleotropic effects due to their capability to regulate multiple genes and pathways. The existence of non-cancerous tissues coupled with the likelihood of triggering toxicity or adverse immune responses increases the level of concern. The efficiency with which these therapies work is severely diminished due to the existence of redundancy and compensatory mechanisms aplenty within the miRNA network when one of the targets is a single miRNA.

The existence of diverse subpopulations of cancer cells, as well as the stromal components within pancreatic tumors, is referred to as tumor heterogeneity. In addition to the challenges already stated, this type of tumor heterogeneity poses additional complications with the expression of varying miRNA profiles within these parts. With these recent advancements come novel ways of tackling the selecting the appropriate miRNA targets und these will prove useful in designing therapies.

Lastly, the lack of uniformity in the techniques used for detecting and measuring miRNAs restricts consistency across various research works. Variations in the steps taken within a single laboratory, such as how samples are prepared, how they are normalized, and how analysis is performed, affect the comparison of results and slow down application in clinical settings.

Innovative approaches to the problems posed by delivery methods, powerful bioinformatics capable of accurately predicting miRNA-mRNA interactions, and well-designed clinical trials assessing safety and efficacy are needed. Only through an integrated approach can therapies targeting miRNAs become practical for treating pancreatic cancer.

## Functional studies and validation techniques

Candidate miRNAs can be selected based on proposed screening criteria, including evidence of aberrant expression in pancreatic cancer, functional evidence of modulation in growth or phenotypic changes, and assessment *in vivo (*84, 165).

Techniques for validating regulatory interactions can be conducted either pre or post functional studies. The reporter methodology involves cloning candidate 3’UTRs with potential binding sites for the miRNA into a vector containing a fluorescent protein encoding sequence. Upon co-transfection with the miRNA expression vector, the protein will be downregulated if the binding is functional, reflected by decreased fluorescent signal (166). miR-145 was validated as a regulator of ten-eleven translocation 2 (TET2) using this technique. A similar approach utilizes a non-coding luciferase reporter gene to measure light production indirectly quantifying miRNA activity (167, 168). miRNAs can also be evaluated through the quantitative assessment of the target mRNA transcript level using RT-qPCR (169). This approach has been used to demonstrate regulation of phosphatase and tensin homolog (PTEN) by miR-21 in pancreatic cancer cells (170). Western blotting can assess expression changes of the corresponding protein target after miRNA modulation, as with miR-27b regulation of Krüppel-like factor 4 (KLF4) in pancreatic cancer (171). Using transcriptome sequencing to compare mRNA levels before and after miRNA modulation can identify candidate targets with significantly altered expression. Bioinformatics predictions can complement functional studies by identifying genes with regulatory sites for the candidate miRNA. Popular algorithms include miranda, picTar, and TargetScan (172, 173). Although these resources have limitations, they are useful for finding and prioritizing candidate targets. Experimental validation of target site functionality is critical, as binding sites may not always represent a direct regulatory relationship.

## Future research directions

As the clinical need for innovative biomarker discovery approaches and applications in pancreatic cancer research and clinical practice rises, the availability of comprehensive biomarker-associated datasets and analytical platforms will encourage new research and clinical explorations emerging from academic, clinical, and industrial communities (174). With an emphasis on implantable microdevice-enabled biomarker discovery, computational pathology, and ex vivo research, Illinois demonstrates how smart biomarker discovery tools drive innovation and collaboration across the bench-to-bedside continuum (175, 176). This invigorating era for pancreatic cancer biomarker discovery will support the accelerated development of the next generation of tools, technologies, and treatments to improve patient outcomes across the cancer continuum. With meeting the clinical needs as the driving force, pancreatic cancer discovery research pivots to the rapidly emerging fields of liquid biopsy, artificial intelligence, exosome, organoid, and microbiome, leveraging interdisciplinary collaborations between researchers with diverse expertise.

The progress and challenges in the discovery, function, and clinical implications of miRNAs in pancreatic cancer are summarized. Focusing on the miRNAs with the most significant findings to date, particular attention is paid to the disruption of miRNA transcription and processing machinery and the experimentally validated role of single miRNAs with oncogenic or tumor-suppressing functions in pancreatic cancer (62). The need to decipher the complex interactions between miRNAs and target genes to better understand the role of miRNAs in pancreatic cancer is also highlighted. Efforts in discovering pan-cancer or biomarker combinations for improved diagnostic accuracy are summarized. A comprehensive pool of candidate biomarkers is provided, consisting of miRNA expression profiles obtained from various biopsy methods that offer insights into the molecular characteristics of pancreatic tumors. Emerging applications of computational methods are also discussed, ranging from simple statistical tests to more sophisticated approaches incorporating machine-learning-based algorithms. The pressing clinical needs for innovative approaches in biomarker discovery are illustrated.

## Conclusion

In conclusion exosomal miRNAs are multifactorial interactions with the pancreatic tumor microenvironment that affect immune modulation, stromal reprogramming, and resistance to therapy. The combination of these two functions as mediators of oncogenic signaling and as a biomarker that can be readily measured, makes them invaluable to the future of pancreatic cancer diagnostics and therapy. Though there has been rapid advancement, methodological inconsistency in the isolation of exosomes, quantification of miRNAnormalization of the reference is a limiting factor to clinical application. Cross-cancer (e.g. miR-21 in pancreatic, breast, and colorectal cancer) comparative studies indicate both commonality of pathways and context-specific regulation, implying that multi-omic validation is necessary. Predictive signatures could be achieved by combining bioinformatics-based miRNA-mRNA network analysis with longitudinal datasets of patients. The next round of research ought to focus on standardized detection platforms, optimization of therapeutic delivery and research into engineered exosomes as miRNA carriers. Clinical translation of exosomal miRNAs is potentially an important breakthrough of the early diagnosis and personalized therapy of pancreatic cancer, and molecular precision is matched to better patient survival. Altogether, this review highlights the translational edge of exosomal miRNAs in pancreatic cancer, which is unlike the previous literature that connects epidemiological urgency with mechanistic and therapeutic knowledge.
